# Plasma interleukin-21 levels and genetic variants are associated with susceptibility to rheumatoid arthritis

**DOI:** 10.1186/s12891-021-04111-0

**Published:** 2021-03-05

**Authors:** Youguo Hao, Lijun Xie, Jing Xia, Zhen Liu, Baoxiu Yang, Minqin Zhang

**Affiliations:** 1 Department of Rehabilitation, Shanghai Putuo People’s Hospital, Putuo People’s Hospital Affiliated to Tongji University, Shanghai, 200060 China; 2 Department of Rehabilitation Medicine, The Third Affiliated Hospital of Sun Yat-sen University, Guangzhou, 510630 China; 3 Southern Medical University, Guangzhou, 510515 Guangdong China; 4 Department of Rehabilitation Medicine, The First People’s Hospital of Foshan, Foshan, 528000 Guangdong China; 5 Department of Chinese Medicine, Wuhan Fourth Hospital, Puai Hospital, Tongji Medical College, Huazhong University of Science and Technology, Wuhan, Hubei, 430033 China

**Keywords:** Rheumatoid arthritis, Interleukin-21, Polymorphism, Chinese

## Abstract

**Background:**

Rheumatoid Arthritis (RA) is a chronic inflammatory condition characterized by autoantibodies development and an elevated spectrum of pro-inflammatory cytokines. Previous reports highlighted a relationship between IL-21and the pathogenesis of RA. Although elevated IL-21 levels have been reported in RA patients, the association of common IL-21 genetic variants with a predisposition to RA development in the Chinese population lacks.

**Materials and methods:**

Five hundred and fourteen Chinese subjects (healthy controls: 303 and rheumatoid arthritis patients: 211) were enrolled in the study. Clinical data of patients were collected from medical records, and patients were treated as per the guidelines. Common single nucleotide polymorphisms in the IL-21 gene (rs907715, rs2221903, rs2055979 and rs6822844) were genotyped by TaqMan SNPs genotyping method. IL-21 level in plasma of RA patients and healthy subjects was measured by ELISA.

**Results:**

The plasma level of IL-21 was significantly higher in subjects with rheumatoid arthritis relative to healthy controls (*p* < 0.0001). A positive correlation was observed between IL-21 level and DAS28 score, indicating the association of the cytokine with the worsening of the disease (Spearman *r* = 0.61, *p* < 0.0001). The prevalence of AA genotype (rs2055979) was significantly higher in RA subjects than in the controls (*p* < 0.0001, χ2 = 34.73, OR = 4.34, 95% CI = 2.623 to 7.219). Furthermore, elevated plasma IL-21 was observed in the rs2055979-AA genotype compared to CC type (*p* < 0.0001).

**Conclusion:**

IL-21 plays a crucial function in rheumatoid arthritis pathogenesis. IL-21 rs2055979 polymorphism is associated with IL-21 plasma levels and is predisposed to RA development in the Chinese population.

## Introduction

Autoimmune diseases are characterized by the unregulated activation of the immune system, which attacks and damages various tissue systems. Although various autoimmune disorders are reported worldwide, rheumatoid arthritis (RA) remained the most prevalent one [1]. RA is a systemic autoimmune disease distinguished by the formation of autoantibodies, inflammation, and enlargement of synovial tissues leading to the destruction of bones and cartilages [2]. The involvement of genetic and environmental factors has been demonstrated with the development of RA [3], and the severity of the diseases depends on several risk factors. Although the disease’s etiology is not fully understood, it is presumed that multiple inflammatory molecules such as cytokines and chemokines play an essential role in disease progression and pathogenesis [4, 5]. Various pro-inflammatory cytokines such as TNF-α, IL-1β, IL-6, and IL-17, have been shown to induce the destruction of cartilages, adjacent bone erosions and increase the severity of the RA pathogenesis [6]. Based on these observations, pro-inflammatory molecules’ regulation has been a crucial targeting approach for developing a possible therapeutic measure against RA. Mainly, inhibition of these inflammatory mediators using the monoclonal antibody approach is of interest that primarily aimed at hindering the synovial inflammation [7]. However, there are many side effects of these monoclonal antibody-based therapies. Additionally, due to prolonged use, these treatment options become ineffective. Therefore, there is always a quest to develop a newer therapeutic approach for the treatment of RA, which can be achieved by venturing into the pathological role of several other inflammatory molecules.

Interleukin-21 (IL-21) cytokine is a member of the IL-2 family mainly produced by CD4^+^ T cells and natural killer T cells (NKT) [8]. However, several reports have also highlighted the production of IL-21 by CD8+ T cells, B cells, macrophages, monocytes, and dendritic cells [9]. IL-21 plays a vital role in the regulation of both innate and adaptive immune systems [10]. Notably, IL-21 controls the differentiation of Th17 cells, B cell activation, and immunoglobulins production [11–13]. The role of IL-21 in the pathogenesis of RA is poorly understood. Elevated levels of IL-21 has been demonstrated in the synovial tissue of RA patients [14, 15]. Further, in the experimental arthritis model, the blockade of IL-21/IL-21 receptor pathways significantly improved disease severity [16], suggesting an essential role of IL-21 in disease pathogenesis. Increased IL-21 has also been associated with higher chances of osteoclastogenesis in humans and mice [15].

In humans, the gene encoding IL-21 is located at the long arm of the fourth chromosome (q26–27). IL-21 gene spans about 8.44 kb of DNA and consists of six exons and five introns. Various single nucleotide polymorphisms (SNPs) have been reported (https://www.ncbi.nlm.nih.gov/SNP/snp_ref.cgi?locusId=59067). Association of different SNPs with autoimmune disorders such as systemic lupus erythematosus [17], graves disease [18], and inflammatory bowel disease [19, 20] have been documented. Various reports have shown a significant association of IL-21 polymorphisms and RA in different populations such as Netherlanders [21], Algerian [22], Columbian [19]. A recent meta-analysis with nine studies [23] demonstrated decreased susceptibility of subjects with IL-21 rs6822844 mutation against RA development. Although the association of IL-21 polymorphisms with RA has been studied in different populations, it has not been explored in the Chinese community. The present study is the first to investigate the possible role of IL-21 polymorphisms in the Chinese cohort.

In the present study, we performed hospital-based case-control research to decipher the role of IL-21 in RA pathogenesis and clinical severity. Furthermore, four common SNPs were genotyped and explored a possible association between IL-21 polymorphisms and predisposition to RA development in the Chinese population.

## Materials and methods

### Study population

Two hundred eleven rheumatoid arthritis patients (156 females and 55 males) were recruited in the present study from January 2018 to December 2019. All patients visited or admitted in the Department of Rehabilitation, Shanghai Putuo People’s Hospital rheumatology division of the hospital and fulfilled the 2010 criteria for American College of Rheumatology/European League against Rheumatism criteria for the classification of RA [24] were enrolled in the study. The mean age of patients was 42.9 ± 13.5 years, and the duration of diseases was 18.3 ± 9.4 months. The exclusion criteria included hypo or hyperthyroidism, diabetes, other autoimmune disorders, chronic liver failure, acute/chronic diarrhea, and congestive heart failure. Three hundred three healthy controls hailing from similar geographical areas, with a mean age of 46.1 ± 18.3 years, were included in the study. RA patients’ various clinical data, such as numbers of swollen and tender joints, disease activity score (DAS 28), and swollen joints count (SJC) were collected from medical records. Further, based on DAS28 scores, patients were sub-grouped into low (DAS 28, < 3.2), intermediate (DAS 28, 3.2–5.1), and high (DAS 28, > 5.1), as per the classification criteria for disease activity by European League against Rheumatism (EULAR) [25]. Different biochemical parameters such as C-reactive protein (CRP), rheumatoid factor (RF), erythrocytic sedimentation rate (ESR), and antibodies to cyclic citrullinated peptides (anti-CCP antibodies) were also examined. All patients were treated with disease-modifying anti-rheumatic drugs (DMARDs) alone or combined with glucocorticoids (GCs). Details of treatments are shown in Table 2. The study was carried out according to the Declaration of Helsinki on ethical principles for medical research involving human subjects [26]. The study protocol was approved by the Institutional Human Ethical Committee of Shanghai Putuo People’s Hospital (PTRMYY20200826), and written informed consent was obtained from each participant.

### Collection of plasma

About 4 mL of intravenous blood was collected from each participant with anti-coagulant at the time of enrollment. Plasma was separated after centrifuging blood at 2500 rpm for 15 min and stored at − 20 °C until further use.

### Isolation of genomic DNA

According to the manufacturer’s instructions, total genomic DNA was isolated from 200 μL of whole blood by using GenElute Blood Genomic DNA Kit (Merck). In brief, about 200 μL of whole blood samples were lysed with lysing solution with proteinase K at 55 °C for 10 min. The lysed cells were loaded in the DNA isolation column and centrifuged at 6500 g for 1 min. Subsequently, the column was washed twice with wash buffer. The membrane-bound DNA was eluted with elution buffer after centrifugation at 6500 g for 1 min. The isolated genomic DNA was stored at − 20 degrees until further use.

### Genotyping of IL-21 polymorphisms

A total of four SNPs (rs907715, rs2221903, rs2055979, and rs6822844) were typed by TaqMan SNPs genotyping method. Predesigned SNP genotyping assays kit were procured from Thermo Fisher Scientific and used to assess enrolled subjects’ genotype. Details of probes are mentioned in Table 1. In brief, a total of 10 μL of the reaction mixture was prepared with 1X TaqMan Genotyping master mix, 1X custom SNP genotyping assay, and 20 ng of DNA from each participant. The reaction cycle was carried out in three steps as follows: step-1, initial heating at 50 °C for 2 min; step-2, heating at 95 °C for 10 min to activate AmpliTaq gold polymerase; step-3, 40 cycles of denaturation at 94 °C for 15 s followed by annealing and extension at 62 °C for 1 min. The fluorescence was read using the allele discrimination program of Applied Biosystems Real-time PCR system (7900HT).

**Table 1:** List of probes used for genotyping of IL-21 polymorphisms

SNPs ID	code	Context sequence
rs907715	C__8949748_10	VIC/FAM-AAAACAGGATTTCCTTGTTTTAACT [C/T]GCATTTATGTGATTACTAGGGAGAT
rs2221903	C__16167441_10	VIC/FAM-ACAGACAATGGGGTTTTGTTTTCTT [C/T]TGTTCTGCAAGCAGCAGAGCTGTGT
rs2055979	C__1597496_20	VIC/FAM-CTAACCATAACAGTTAAACAAGGTG [C/A]ATGAGATGCTAGAAATGTATGTTTT
rs6822844	C__28983601_10	VIC/FAM-CCTGTCTCGCTCTCCATAGCAAAAA [G/T]AGAGGACTCTTTTCATGTTGCCACT

### Enzyme-linked Immunosorbent assay

Plasma level IL-21 was measured in patients and controls using human IL-21 Duo Set ELISA kit (R&D Systems, Inc., USA) according to the manufacturer’s instructions. All plasma samples were measured in duplicate, and the average absorbance value was recorded for a study subject. Furthermore, various autoantibodies such as Anti-cyclic citrullinated peptide (Anti-CCP: Euroimmune, Germany), anti rheumatoid factors (IgG and IgM: Abnova, Germany) were quantified by ELISA according to the instructions of the manufacturer’s.

### Statistical analysis

The statistics analysis was performed by GraphPad Prism version 8.3.0 (GraphPad Software, Inc., La Jolla, CA, USA). The distribution of variables was tested by the D’Agostino-Pearson omnibus normality test. Based on the normality test result, differences in IL-21 levels in RA patients and HC were compared by Mann Whitney U test. Other comparisons with more than two groups were performed with analysis of variance (ANOVA) followed by Tukey’s post-test. Further, the relationship between the IL-21 and DAS 28 scores was conducted by Spearman’s correlation test. Genotype and allele frequency in RA patients and healthy controls were compared by Chi-square (χ^2^) test. A *p*-value of less than 0.05 was considered statistically significant.

## Results

### Baseline characteristics of enrolled subjects

Baseline characteristics of rheumatoid arthritis patients and healthy controls are shown in Table 2. As demonstrated earlier, the RA is most frequent in females compared to males. In our studied cohort, female patients were 2.83 folds higher chance of having RA compared to males. Biochemicals parameters such as ESR and CRP levels were significantly elevated in RA patients compared to healthy controls. Significantly, results for subgrouping of patients based on DAS 28 score revealed that 30.3% of subjects had low disease activity (DAS 28, < 3.2), whereas, 36.9% of subjects had medium (DAS 28, 3.2–5.1) and the remaining 32.8% patients showed a high disease activity (DAS 28, > 5.1). On screening of RA patients’ rheumatoid factors, about 63% of patients were found positive for RF, and 62% of patients had antibodies to cyclic citrullinated peptides (CCP).

**Table 2:** Baseline characteristics of study subjects

Parameters	Rheumatoid arthritis patients	Healthy controls
Total numbers	211	303
Gender (F/M)	156/55	210/93
Age (Mean ± SE)	42.9 ± 13.5	46.1 ± 18.3
Disease duration (Months)	18.3 ± 9.4	NR
Swollen joint counts (0–28)	7.0	NR
Tender joint counts (0–28)	13.0	NR
DAS28 score (%)		NR
< 3.2	30.3	
Between 3.2–5.1	36.9	
> 5.1	32.8	
SJC out of 66	9.4 ± 6.3	NR
ESR (mm at 1st hour)	37.6 ± 21.4	17.8 ± 11.2
CRP (mg/mL)	18.9 ± 22.4	1.19 ± 13.2
RF positivity (%)	63	NR
Anti-CCP antibody positive (%)	62	NR
Treatment Details		NR
Methotrexate	52%	
Sulphasalazine	46%	
Hydroxychloroquine	5%	
Leflunomide	2%	
Tocilizumab	12%	
Adalimumab	6%	
Infliximab	5%	
Prednisone	12%	

Data are presented as either in number, mean ± SE, or percentage

*DAS* Disease Activity Score, *SJC* Swollen Joint Count, *ESR* Erythrocytic Sedimentation Rate, *CRP* C reactive protein, *RF* Rheumatoid Factor, *CCP* cyclic citrullinated protein, *NR* Not required

### RA patients displayed higher plasma IL-21 levels

**Fig. 1:**
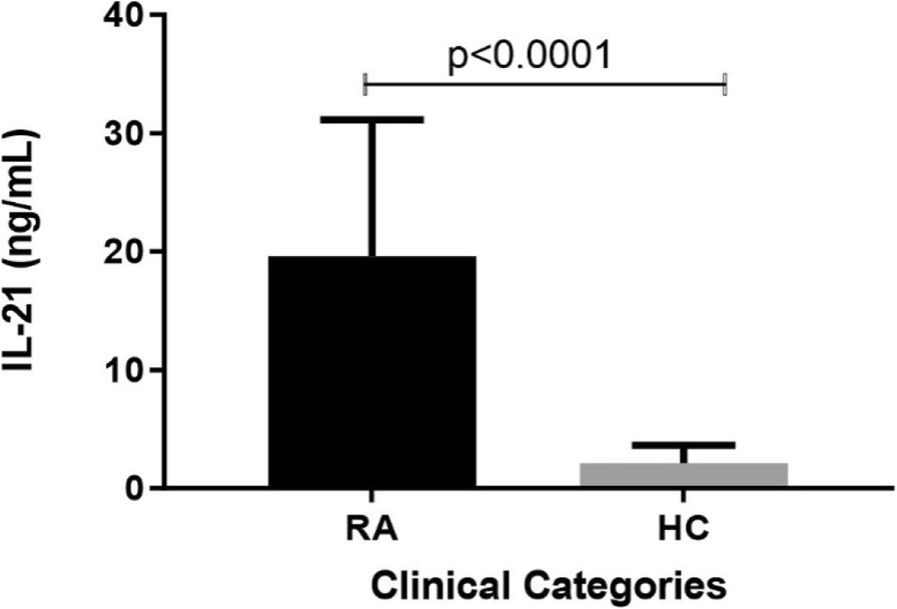
Plasma IL-21 levels in subjects with RA (*n* = 211) and healthy controls (*n* = 303). Data represented the mean IL-21 ng/mL ± SD and were compared by Mann Whitney U test. *P* < 0.05 was deemed to be significantly positive

Plasma levels of IL-21 in RA patients and healthy controls were quantified by ELISA, and results are shown in Fig. 1. RA patients (19.6 ± 0.79 ng/mL) displayed significantly higher levels of plasma IL-21 compared to healthy controls (2.12 ± 0.08 ng/mL) (*p* < 0.0001).

### Association of plasma IL-21 levels and DAS28 scores

**Fig. 2:**
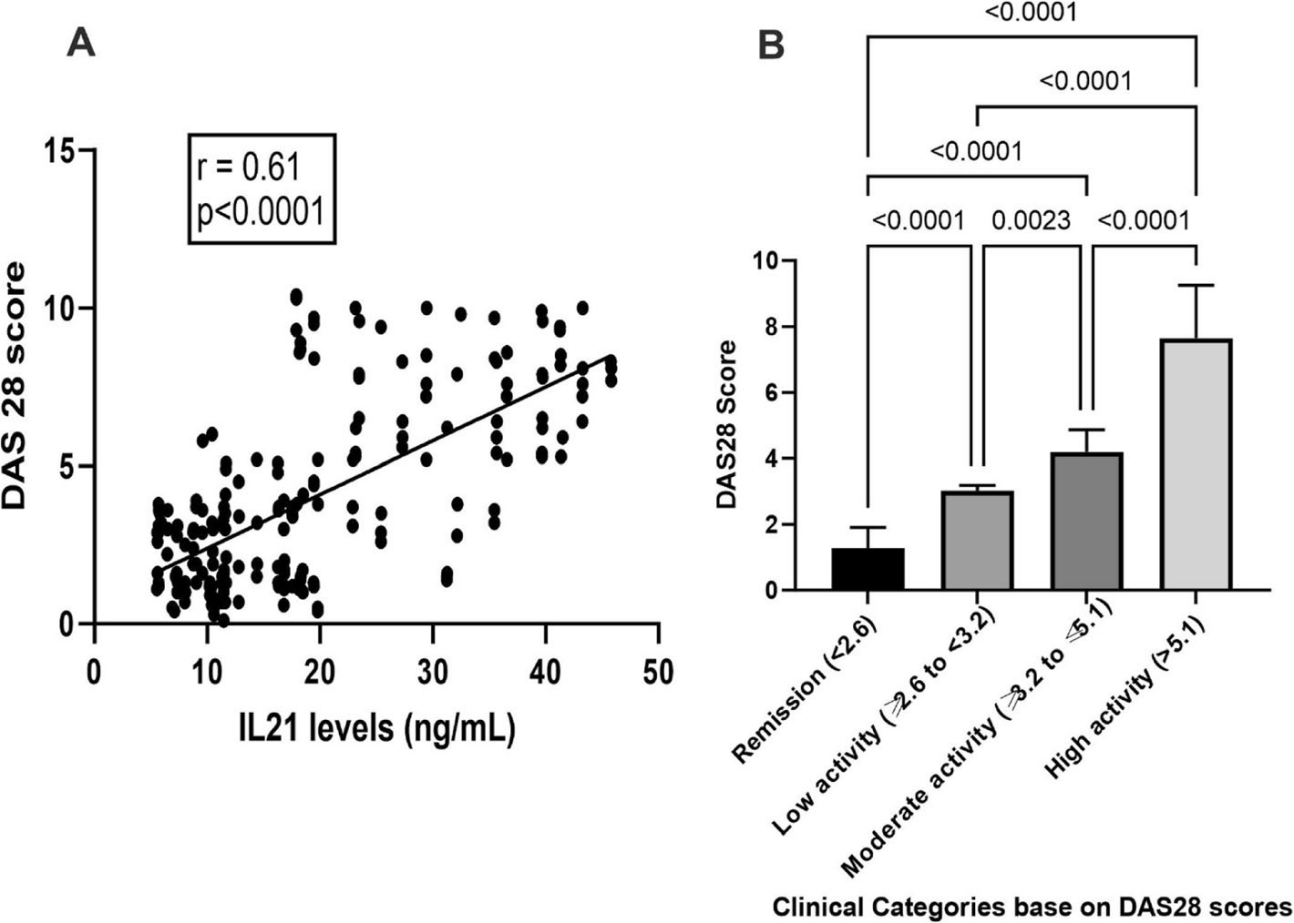
Correlation between plasma IL-21 level with DAS score. IL-21 levels were measured by ELISA in all RA patients and correlated with the DAS score of RA patients. A positive correlation was observed among IL-21 levels and DAS scores (**a**). Further, RA subjects were subcategorized in to four groups based on DAS scores such as remission [DAS 28 < 2.6 (*n* = 52)], low activity [DAS 28, ≥2.6 to < 3.2 (*n* = 12)], medium activity [DAS 28, ≥3.2 to ≤5.1 (*n* = 79)] and high activity [DAS 28, > 5.1 (*n* = 68)]. Data represented the mean of IL-21 levels (ng/mL) ± SD and were compared using ANOVA with Tukey’s post-test. A *P*-value of less than 0.05 was taken as statistically significant

As the DAS28 scores represent the disease severity of rheumatoid arthritis patients, we hypothesized a possible correlation between DAS28 scores and plasma levels of IL-21. Spearman rank coefficient analysis revealed a significant positive correlation between plasma IL-21 levels and DAS28 scores (spearman *r* = 0.61, *P* < 0.0001) (Fig. 2a).

RA patients were further categorized into four subgroups based on DAS28 scores. As shown in Fig. 2b, RA patients with higher disease activity scores (DAS28 > 5.1) had higher mean plasma IL-21 levels compared to those with medium (*p* < 0.0001) and low disease activity scores (*p* < 0.0001) and remission (*p* < 0.0001). Furthermore, a significant difference in mean levels of plasma IL-21 was observed among the lower and intermediate disease activity group (*p* < 0.0001) (Fig. 2b).

IL-21 has been linked with increased follicular T cells, elevated B cell activation, proliferation, and production of antibodies [27]. Further, IL-21 levels are positively correlated with disease activity scores in rheumatoid arthritis patients [27]. However, no significant correction was observed among IL-21 and anti-CCP, RF-IgG, and RF-IgM levels in the present study (data not shown).

### Distribution of IL-21 polymorphisms in the healthy Chinese population

A total of 303 healthy Chinese subjects were genotyped for four common SNPs (rs907715, rs2221903, rs2055979, and rs6822844) TaqMan genotyping method. All subjects were having major genotype (GG) for rs6822844 polymorphism [17]. As shown in Table 3, heterozygous mutants were more frequent in rs907715 and rs2055979 polymorphism, followed by wild type and homozygous mutant. Further, for rs2221903 polymorphism, the wildtype remained highly prevalent compared to heterozygous (23%) and homozygous mutant (2%). Distribution of genotypes for three SNPs were in Hardy-Weinberg Equilibrium (HWE) (rs907715: χ^2^ = 0.01, *p* = 0.90, rs2221903: χ^2^ = 0.04, *p* = 0.82, rs2055979: χ^2^ = 0.18, *p* = 0.66).

**Table 3:** Prevalence of IL-21 polymorphisms among controls and RA patients

Polymorphisms	Genotype or Allele	HC (*n* = 303)	RA (*n* = 211)	*P*-value	χ^2^ value	OR (95% CI)
**rs907715 C > T**	Genotype					
	CC	91 (30)	68 (32)	1		ref
	CT	151 (50)	103 (49)	0.656	0.197	0.912 (0.613 to 1.366)
	TT	61 (20)	40 (19)	0.613	0.254	0.877 (0.525 to 1.444)
	CT + TT	212 (70)	143 (68)	0.596	0.280	0.902 (0.622 to 1.319)
	Allele					
	C	333 (55)	239 (57)	1		ref
	T	273 (45)	183 (43)	0.592	0.286	0.934 (0.724 to 1.202)
**rs2221903 T > C**						
	TT	227 (75)	154 (73)	1		ref
	TC	70 (23)	49 (23)	0.883	0.021	1.032 (0.676 to 1.581)
	CC	6 (2)	8 (4)	0.211	1.561	1.965 (0.658 to 5.779)
	TC + CC	76 (25)	57 (27)	0.622	0.242	1.106 (0.738 to 1.635)
	Allele					
	T	524 (86)	357 (85)	1		ref
	C	82 (14)	65 (15)	0.399	0.711	1.163 (0.822 to 1.655)
**rs2055979 C > A**	Genotype					
	CC	118 (39)	53 (25)	1		ref
	CA	145 (48)	80 (38)	0.341	0.906	1.228 (0.811 to 1.888)
	AA	40 (13)	78 (37)	< 0.0001	34.73	**4.342 (2.623 to 7.219)**
	CA + AA	185 (61)	158 (75)	0.001	10.71	**1.901 (1.301 to 2.796)**
	Allele					
	C	381 (63)	186 (44)	1		ref
	A	225 (37)	236 (56)	< 0.0001	35.53	**2.149 (1.662 to 2.766)**

Note: Data of HC and RA are in the number of subjects (%) format, *HC* healthy controls, *RA* rheumatoid arthritis patients

### Association of IL-21 rs2055979 polymorphism with susceptibility to RA

To test whether common genetic variants in the IL-21 gene are associated with predisposition to rheumatoid arthritis development, we genotyped rs907715, rs2221903 rs2055979 polymorphism in 211 RA patients and 303 healthy controls. As shown in Table 3, the prevalence of homozygous mutant (AA) of rs2055979 polymorphism was significantly higher in RA patients compared to healthy controls (*p* < 0.0001, χ^2^ = 34.73, OR = 4.342). The frequency of mutants (CA + AA) was also higher in RA than in controls (*p* = 0.001, χ^2^ = 10.71, OR = 1.901). Furthermore, the mutant allele (A) was even more frequent in patients than healthy controls (*p* < 0.0001, χ^2^ = 35.53, OR = 2.149), indicating an essential genetic susceptible factor on predisposition to RA development.

### Functional relevance of IL-21 rs2055979 polymorphism

**Fig. 3:**
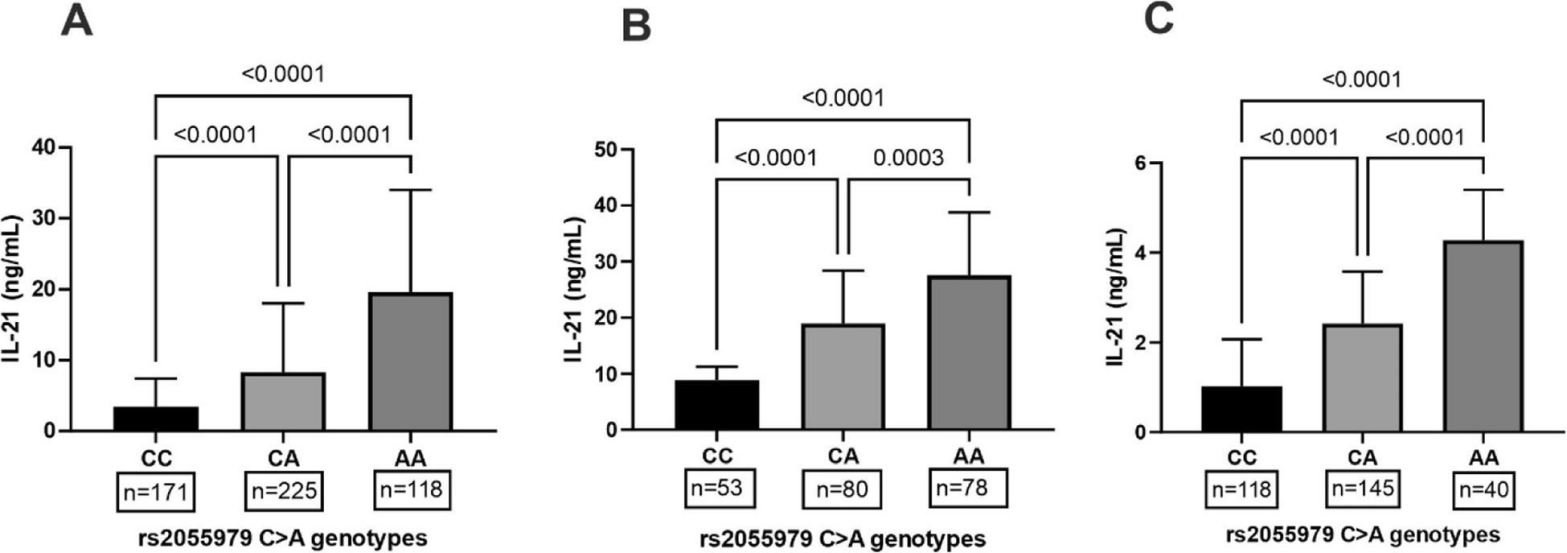
Association of IL-21 rs2055979 polymorphism with plasma IL-21 levels. Plasma levels of IL-21 were quantified by ELISA in both RA patients (211) and healthy controls (*n* = 303). The distribution of Plasma IL-21 in the different genotypes of rs2055979 polymorphism revealed higher levels in AA genotype compared to other genotypes (**a**). A similar observation was noticed in RA patients (**b**) and healthy controls (**c**). Data are represented in mean ± SD, and plasma levels of IL-21 in different genotypes were compared with the Kruskal-Wallis test accompanied by Tukey’s post-test. *P* < 0.05 was deemed to be statistically significant

Plasma levels of IL-21 in RA patients and healthy controls were analyzed among different genotypes of IL-21 polymorphisms (rs907715, rs2221903, and rs2055979) to investigate the possible association plasma IL-21 levels. As shown in Fig. 3a, the AA genotype of rs2055979 polymorphisms had higher plasma levels of IL-21 than other genotypes, i.e., CA demonstrated intermediate levels, and CC had the lowest levels of plasma IL-21. Interestingly, similar observations were noticed when the association of IL-21 rs2055979 polymorphism was analyzed in RA patients (Fig. 3b) and healthy controls (Fig. 3c). For other studied SNPs (rs907715 and rs2221903), no significant association between genotypes and plasma levels of IL-21 was observed (data not shown).

### Association of IL-21 rs2055979 polymorphism with DAS28 scores

**Fig. 4:**
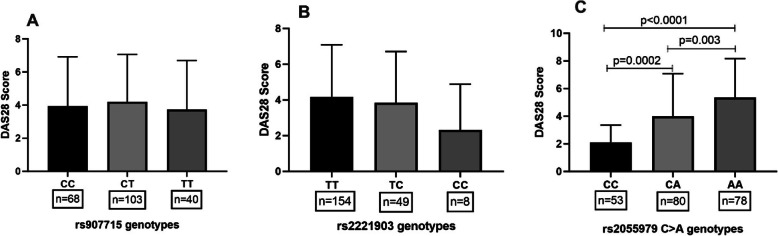
Association of IL-21 gene polymorphisms (rs907715, rs2221903, and rs2055979) with DAS28 scores. The DAS28 score was comparable in different genotypes of rs907715 (**a**) and rs2221903 (**b**) polymorphism. A significant difference was noticed in rs2055979 polymorphism with DAS-28 scores. AA genotype was associated with higher DAS28 scores compared to other genotypes (**c**). Data are represented in the mean ± SD, and plasma levels of IL-21 in different genotypes were compared with ANOVA accompanied by Tukey’s post-test. *P* < 0.05 was deemed to be statistically significant

As DAS 28 and plasma levels of IL-21 were correlated; further, we analyzed the possible association of IL-21 polymorphisms with DAS28 scores. As shown in Fig. 4c, we observed a significant association between IL-21 rs2055979 polymorphism with DAS28 scores: subjects with AA genotyped had higher DAS28 scores than CA and CC genotypes. However, such association was not observed in rs907715 and rs2221903 polymorphisms (Fig. 4a and b).

## Discussion

The role of different cytokines in mediating the pathogenesis of rheumatic diseases has been well documented. Prior reports suggested that some cytokines secreted by Th1, Th2, and Th17 cells have been designated as potent biomarkers in RA’s pathogenesis [28]. Studies in Chinese RA patients are limited. A report during 2011–2012 indicated the significance of chemokines, pro and anti-inflammatory cytokines RA [29]. In the Chinese population, however, the role of IL-21 in RA pathogenesis has never been critically studied.

In the present investigation, we observed a significantly elevated plasma IL-21 in Chinese patients with RA compared to healthy controls. These results are corroborated with previous reports. An earlier hospital-based case-control study in Chinese patients demonstrated higher serum IL-21 levels than healthy controls [30]. Similarly, in a longitudinal study in patients with early-stage RA, IL-21 level was upregulated in diseased subjects compared to controls [31]. All of these findings, including our results, indicated the possible function of IL-21 in the advancement of RA pathogenesis. Nevertheless, controversial results do still occur. There was no substantial difference in serum IL-21 level between subjects with recent RA onset and healthy controls in a study by Sglundaet al [32]. Furthermore, in rheumatoid arthritis patients with higher disease activity (DAS28 > 5.1) and healthy control levels, IL-21 levels were also comparable [32]. Although the exact reason for such discrepancy in data is not known, the use of fewer patients (*n* = 51) in the given study may be a contributing factor.

An independent study [32] have highlighted comparable IL-21 levels between high disease activity (DAS28, > 5.1) RA patients and healthy subjects. On the contrary, we observed a significantly higher level of IL-21 in the patient group with DAS 28 > 5.1 compared to the other three groups (DAS28 < 2.6, DAS28 ≥ 2.6 to < 3.2, and DAS28 3.2–5.1) and healthy controls. In line with these findings, higher plasma levels of IL-6 and IFN-α were recorded in rheumatoid patients with higher disease activity than those with lower DAS28 scores [33].

In our current research, a steady rise in plasma IL-21 in the higher disease activity of the patients was observed. This finding led us to investigate further the possible link between the plasma IL-21 levels and DAS 28 scores. A positive association between IL-21 and DAS28 was observed, corroborating earlier observations [27, 32]. However, another study found no connection between IL-21 and DAS 28 in 126 Chinese RA patients [30].

The role of IL-21 in the pathogenesis of rheumatoid arthritis is well investigated. IL-21 is mostly secreted by T helper 17 cells (Th17), follicular T helper cells (Tfh), and natural killer cells (NKT) [34]. IL-21 facilitates the activation of B cells, NK cells, and the production of antibodies. The IL-21 receptor (IL-21R), which recognizes IL-21 as a ligand, is highly expressed on CD4+ T cells on macrophages and dendric cells in RA patients [35]. Cells expressing IL-21R recognizes IL-21 and respond through MAPK, PI3K/AKT, and JAK-STAT pathways [34]. Several reports in the mouse model also further strengthen the importance of IL-21 in RA. Administration of IL-21 receptor Fc fusion protein (IL-21RFc), a neutralizing agent of IL-21 in the arthritis model, significantly diminished IL-6 and IL-17 [16]. Furthermore, injection of IL-21RFc in collagen-induced arthritis significantly reduced the disease progression [16]. Besides, RA synovial cell culture with IL-21RFc significantly reduces the production of TNF-α, IL-6, and IL-1β [36].

The association of IL-21 polymorphisms with a predisposition to RA development has been extensively investigated in different populations. In most of the research, the role of rs6822844 polymorphism was investigated to find a potential link with the susceptibility to the development of RA. Reports including RA patients from different geographical regions showed the protective role of the rs6822844 variant against RA development in the Netherlands [21], Algerian [22], Columbian [19] population. The latest meta-analysis further strengthens individual case-control observation [23]. However, both patients and controls were wild types for rs6822844 polymorphism, similar to an earlier study in the Chinese population [17]. Collectively these observations indicate the absence of rs6822844 variants in the Chinese population.

In this study, we observed a significant role in rs2055979 polymorphism with RA predisposition. Subjects carrying the genotype of AA had a 4.34-fold higher susceptibility to RA. However, the distribution of other common polymorphisms among healthy controls and RA patients was comparable. Earlier research in Chinese systemic lupus erythematosus patients recorded similar observations: rs2055979 was correlated with susceptibility, whereas rs907715 and rs2221903 polymorphisms did not play a significant role [17]. Similarly, in an earlier study, rs907715 polymorphism failed to associate RA susceptibility in the Australian population [37]. Besides, an essential functional significance of rs2055979 polymorphism was noted in the present report: subjects with AA genotype had higher plasma IL-21 than those with CC genotype. Interestingly, heterozygotes demonstrated intermediate levels of Il-21. Similar association trends have been observed in both healthy control and RA patients. In line with our findings, an earlier study showed a substantial difference in AA and CC genotype plasma IL-21 levels. However, differences between heterozygous and wild or homozygous mutants could not be detected, likely due to the limited sample size. The mechanism of how the AA genotype is correlated with higher IL-21 levels is not understood. The SNP rs2055979 is located in the intronic region and may have an impact on the splicing process [38].

Although we have included a larger cohort of healthy controls and RA patients and demonstrated the importance of IL-21 in the present investigation, the present report has several limitations. First, all patients included in the study were not naïve, and most of them were treated before enrollment in the study, which may affect the patients’ clinical parameters. Second, in the present report, we have considered only four SNPs in the IL-21 gene. Thus, other functional SNPs in the IL-21 gene may be studied in the future. Third, the samples enrolled in the investigation were hailed from Shanghai and adjacent areas. It would be interesting to replicate the study in other populations.

In conclusion, IL-21 plasma levels are increased in patients with rheumatoid arthritis associated with disease severity. Furthermore, IL-21 (rs2055979) mutant is associated with elevated IL-21 plasma levels and is predisposed to RA development. However, further studies are required in different populations to validate our findings.

## Data Availability

Data will be available upon request to the corresponding author.
